# 

**Published:** 1878-01-20

**Authors:** 


### ORIGINAL AND SELECTED ARTICLES.

Dyspepsia; by Fred. King, M.D. .... 1.
Poisoning with Tainted Meat—by L. B. Anderson, M.D. .......... 3
Metrorrhagia; Hemorrhage from the Womb—by I. J. Goss, M.D. ...... 5
A Case of Addison's Disease. ......... 6
A Case of Arsenical Poisoning Treated with Dialysed Iron—by Thos. B. Reed, M.D ........ 8
Epistaxis. ........ 8
The Bath of Decreasing Temperature in the Treatment of Typhoid Fever—by H. M. Garten, M.D... 9
Breech Presentations—by J. E. Clark, M.D. ........ 11
Irrigation of the Large Intestine—by C. W. Dulles, M. D. ........ 12

### ABSTRACTS AND GLEANINGS.

Treatment of Hysteria, 14; Aromatic Syrup of Liquorice, 15; Santonin, 16; Combined use of Chloroform and Morphia, 16; Chloroform Hallucination and Alledged Rape, 17; The Employment of Anæsthetics in Labor, 17; The Treatment of Ozæna, 18; Ulceration of the Os Uteri, 19; Protracted Labor, 19; The Treatment of Sciatica, 20; Extraction of Quinamia from Red Bark, 20; The Poisonous Dose of Castor Oil Seeds, 20; Antagonism of Belladonna and Opium, 20; The Use of the Trephine in Depressed Fractures of the Skull, 21; Washing Out the Bladder; 21; Conium, 22; Salicylic Acid, 22; Chloral Hydrate, 23; Ergot, 23.

### PRACTICAL NOTES AND FORMULÆ.

Note from Dr. O. E. Newton on How to Give Podophyllin, 24; Permanent Cure for Costiveness, 24; Note from Dr. O. E. Newton on Removal of Placenta, 24; Paralysis of Diphtheria, 24; Pseudo Hypertrophic Paralysis, 25; Night Cramp, 25; Collodion Paints, 25; Inflammation of the Os, or in Vaginismus, 25; Inter Uterine Injections without the Use of Speculum, 25; To Make Ice Water, 25; Laryngeal Affections and Catarrh, 26; Delirium Tremens, 26; Cheap Tonic, 26; For Tape Worm, 26; Vermifuges, 26; Whooping Cough, 26; Early Rupture of Membranes, 27; Ovarian Tumors, 27; Sore Nipples, 27; Syphilitic Rheumatism, 27; Mortality in Philadelphia, 27; Membranous Croup, 27; Kerosene Liniment, 28; For Bronchitis, 27; Quinine for Hypodermic Use, 27.

### SCIENTIFIC ITEMS.

Edison's Phonograph, 28; Ancient Sciences, 28; Sun's Distance, 28.

### EDITORIAL AND MISCELLANEOUS

To Our Subscribers, 29; Our Journal for 1878, 29; Our Publishing House, 29; Lactopeptine, 29; Parvules, 29; Dialysed Iron, 29; Spermatic Truss, 29; Our Co-Laborers, 29; Abingdon Academy of Medicine, 30; Book Notices, 31; Important Errors, 32; Messrs. Bullock & Crenshaw's Rejoinder to W. R. Warner & Co., 32.

